# Pneumoparotitis

**DOI:** 10.1590/S1808-86942011000600020

**Published:** 2015-10-19

**Authors:** Diego Ferrasso Zuchi, Paola Conrad da Silveira, Cristiano de Oliveira Cardoso, Wilson Madeira de Almeida, Carlos Jader Feldman

**Affiliations:** 1 Resident Physician in Radiology and Image Diagnostics - Instituto de Cardiologia do RS/ FUC; 2 Radiologist - SIDI Medicina por imagem; 3 MSc in Health Sciences: Cardiology, Hemodynamics; 4 MD. Cardiovascular Radiologist. Head of the Radiology and Image Diagnostics at SIDI; 5 MD. Cardiovascular Radiologist. Head of the Radiology and Image Diagnostics at SIDI

**Keywords:** diagnostic imaging, parotid gland, ultrasonography

## INTRODUCTION

The pneumoparotid term, first described in 1865 by Hyrtl^1^, defines the presence of air within parotid system (gland and Stensen duct).

The mechanism causing the reflux of air through the Stensen duct is basically explained by an excessive intraoral pressure, associated to a defect in the preventive mechanisms of reflux. Saliva and air bubbles may be seen coming from the Stensen duct during gland massage/palpation, and crackling noises. Gland edema and local pain make up the classic clinical picture^2^.

## CASE REPORT

Patient: a 50-year-old woman started three years ago with sudden ear pain on the left side. Evaluation showed that the pain was not coming from the ear, but rather from the left parotid gland.

An ultrasound exam of the salivary glands showed a solid nodule, with small peripheral calcifications on her left parotid gland. Punction and histology of the nodule showed numerous typical lymphocytes, without malignant cells.

Her pain worsened together with the edema in her left cervical and mandibular regions, she had sialorrhea with drainage of a purulent secretion through the left parotid duct.

**Figure 1:**
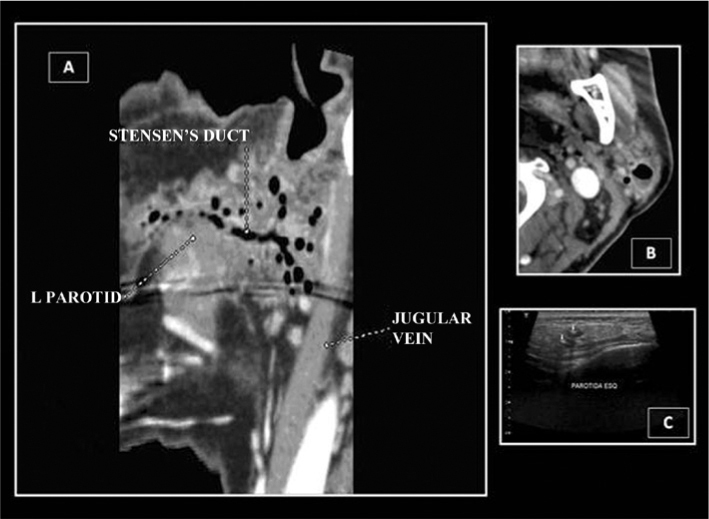
Radiological aspect of the pneumoparotitis seen through CT scan (A and B) and ultrasound (C).

A neck CT scan found a scalloped lesion with air-fluid level, indicative of an abscess in the lower left parotid pole. Multiple air-filled small cavitations within the ductal tree and in the Stensen duct, indicative of an inflammatory process with an abscess (pneumoparotitis) (Figures 1A and 1B). Another ultrasound scan of the left parotid gland confirmed the CT findings (Fig. 1C).

Treatment was indicated with sulphametoxazole/trimethoprim for 14 days, together with local symptomatic measures. There was an improvement in the purulent secretion; nonetheless, there was a mild persistence of the temporary local edema and otalgia in the 1 year follow up.

## DISCUSSION

The presence of air inside the parotid gland has been called many names: pneumoparotitis, pneumoparotiditis, pneumoparotis, pneumosialadenitis, pneumatocele and glandulae parotis^1^. The mechanism through which air may penetrate the Stensen duct is not explained only by the increase in intraoral pressure^2^. Other predisposing factors have been associated with anatomical abnormalities, including a pathological Stensen duct; hypotonia of the buccinator muscle, hypertrophy of the masseter muscle and temporary obstruction of the Stensen duct by mucous^1^, ^2^, ^3^, ^4^.

The parotid glands are not usually palpable. Swollen glands suggest disease. Infections, autoimmune processes, malnutrition, endocrine disorders, drugs and duct obstruction are the most common causes. Pneumoparotitis is a rare cause of parotid gland edema.

Clinical treatment is the initial approach, and surgery is reserved for symptomatic and irreversible cases^1^.

## FINAL REMARKS

Although having air in the parotid gland or in the ducts is mandatory, the diagnosis of pneumoparotitis can be made clinically when there are predisposing factors, such as a bulged and crackling gland, and when air bubbles are seen coming from the duct hole^2^.

Common head and neck x-ray scans may show air inside the Stensen duct and the parotid gland, in severe cases there is subcutaneous emphysema. Nonetheless, regular x-rays do no rule out the disease^5^.

Sialography may find sialectasis, radiolucent stones, ductal stenosis and radiolucent areas inside the intraductal contrast, which may represent air pockets entrapped by the contrast injection^3^. Its association with MRI is a valuable tool in the investigation of recurrent pneumoparotitis^5^.

Ultrasound is deemed useful for diagnosis and follow up purposes^5^. Nonetheless, CT scan is the preferred diagnostic and follow up approach, since it detects air inside the gland and determines its extension through the ducts^5^.
